# Setting strategy for system change: using concept mapping to prioritise national action for chronic disease prevention

**DOI:** 10.1186/s12961-017-0231-7

**Published:** 2017-08-08

**Authors:** Sonia Wutzke, Nick Roberts, Cameron Willis, Allan Best, Andrew Wilson, William Trochim

**Affiliations:** 1 The Australian Prevention Partnership Centre, Level 13, Building 10, 235 Jones, Ultimo, NSW 2007 Australia; 2 Menzies Centre for Health Policy, D02 Victor Coppleson Building, University of Sydney, Camperdown, NSW 2006 Australia; 3 NSW Ministry of Health, 73 Miller St, North Sydney, NSW 2060 Australia; 4 InSource Research Group, 6975 Marine Drive, West Vancouver, BC V7W 2T4 Canada; 5 Cornell University, 435 Kennedy Hall, Ithaca, NY 14853 United States of America

**Keywords:** Prevention, Chronic disease, Public health, Health policy, Systems change, Concept mapping

## Abstract

**Background:**

Chronic diseases are a serious and urgent problem, requiring at-scale, multi-component, multi-stakeholder action and cooperation. Despite numerous national frameworks and agenda-setting documents to coordinate prevention efforts, Australia, like many countries internationally, is yet to substantively impact the burden from chronic disease. Improved evidence on effective strategies for the prevention of chronic disease is required. This research sought to articulate a priority set of important and feasible action domains to inform future discussion and debate regarding priority areas for chronic disease prevention policy and strategy.

**Methods:**

Using concept mapping, a mixed-methods approach to making use of the best available tacit knowledge of recognised, diverse and well-experienced actors, and national actions to improve the prevention of chronic disease in Australia were identified and then mapped. Participants (ranging from 58 to 78 in the various stages of the research) included a national sample of academics, policymakers and practitioners. Data collection involved the generation and sorting of statements by participants. A series of visual representations of the data were then developed.

**Results:**

A total of 95 statements were distilled into 12 clusters for action, namely Inter-Sectoral Partnerships; Systems Perspective/Action; Governance; Roles and Responsibilities; Evidence, Feedback and Learning; Funding and Incentive; Creating Demand; Primary Prevention; Social Determinants and Equity; Healthy Environments; Food and Nutrition; and Regulation and Policy. Specific areas for more immediate national action included refocusing the health system to prevention over cure, raising the profile of public health with health decision-makers, funding policy- and practice-relevant research, improving communication about prevention, learning from both global best-practice and domestic successes and failures, increasing the focus on primary prevention, and developing a long-term prevention strategy with an explicit funding commitment.

**Conclusions:**

Preventing chronic diseases and their risk factors will require at-scale, multi-component, multi-stakeholder action and cooperation. The concept mapping procedures used in this research have enabled the synthesis of views across different stakeholders, bringing both divergent and convergent perspectives to light, and collectively creating signals for where to prioritise national action. Previous national strategies for chronic disease prevention have not collated the tacit knowledge of diverse actors in the prevention of chronic disease in this structured way.

**Electronic supplementary material:**

The online version of this article (doi:10.1186/s12961-017-0231-7) contains supplementary material, which is available to authorized users.

## Background

Internationally, chronic diseases are recognised as a serious and urgent population health problem [1]. Chronic diseases and their risk factors are complex problems, influenced by biological, social, physical, cultural and economic factors that combine in non-linear ways to shape individual choices, exposure, risk factor development, and disease incidence and progression. Despite their complexity, the major chronic diseases are largely preventable through interventions that reduce tobacco use, high body mass, alcohol use, physical inactivity and high blood pressure [2]. Given the complex nature of these risk factors and the chronic diseases they cause, effective preventive interventions are likely to require several components, delivered at multiple levels, and tailored to changing contexts and circumstances [3]. Examples of these ‘complex interventions’ include comprehensive tobacco control interventions, community-based obesity prevention strategies, and multi-component healthy eating and nutrition initiatives.

In Australia, there has been a stream of national strategies that attempt to coordinate prevention efforts, including how complex interventions are designed, delivered and improved. The origins of these actions was most visible when Australia became a signatory to the Global Strategy for Health for All by the Year 2000 in 1981 [4], which led to the establishment of the Better Health Commission in 1985 and its landmark report in 1986 ‘Looking Forward to Better Health’ [5]. The follow-on Health for All Committee released its ‘Health for all Australians’ report in 1988 [6] and the first set of goals and targets for a healthier Australia was released in 1993 [7]. In 1994, the Australian Health Ministers’ Advisory Committee released its report ‘Better Health Outcomes for Australians’ [8] and, in 1996, Health Ministers agreed to the first set of National Health Priority Areas, most of which were chronic diseases. The National Public Health Partnership established in 1996 had a broader remit than chronic disease, but produced a number of supporting technical documents as well as specific strategies, for example, in nutrition [9].

In the immediate past decade the cascade of strategies have included the development of a National Chronic Disease Strategy [10]; the establishment of the Council of Australian Government’s National Partnership Agreement on Preventive Health, which saw the largest single investment in chronic disease prevention in the history of Australia [11]; the establishment of the National Preventative Health Taskforce and the release of their subsequent report ‘Australia: The Healthiest Country by 2020’, including an overview and roadmap for action to improve the prevention of chronic disease [12]; and ‘Taking Preventative Action’, the Commonwealth Government’s response to the National Preventative Health Taskforce Strategy [13]. Australia, like other Member States of WHO, has also committed to the WHO’s Global Action Plan for the Prevention and Control of Noncommunicable Diseases (also referred to as chronic diseases) [1]. This ‘road map and a menu of policy options’ aims to substantially reduce the burden of premature mortality from chronic diseases through action on nine voluntary global targets measured by 25 indicators of performance, including a 25% relative reduction in premature mortality from chronic diseases by 2025.

Despite these national frameworks and agenda-setting documents, there is limited evidence that Australia will meet many of WHO’s 25 performance indicators by 2025 [14]. The reasons for this are many and varied, including challenges of implementation and a lack of investment in preventive action [14]. We also posit that Australia’s efforts to significantly impact on chronic disease are hampered by the lack of a common, united agenda across the various sectors that have the potential to influence chronic disease. Further, the many strategic documents overall identify broad directions and high level goals rather than specific and targeted actions. Finally, we are not aware of any attempt to identify clear priorities to advance prevention that differentiate between importance and feasibility of implementation; two notions that often are not well aligned. To advance and better target efforts to improve the prevention of chronic disease, this study sought to surface a priority set of both important and feasible targeted action domains, collated through the tacit knowledge of recognised, diverse and well-experienced actors.

## Methods

### Concept mapping

There is growing interest in the value of systems thinking and systems methods for tackling complex public health issues like chronic disease [15]. Systems approaches acknowledge that the causes of complex problems like chronic diseases are numerous, varied, dynamic and interconnected. Systems approaches recognise and intervene in the root causes of a problem – the patterns of behaviour, the underlying structure and the beliefs (mental models) of the people and organisations who are both responsible for creating that complex issue and also in positions to change the issue.

In this research, we used an established systems method, concept mapping, to surface the tacit knowledge and beliefs of diverse actors who can influence the prevention of chronic disease. Concept mapping is a mixed methods approach with qualitative procedures to generate thoughts across a group on a topic of interest, followed by quantitative methods to synthesise and represent the group’s ideas visually in a series of maps [16, 17] as well as additional qualitative methods to interpret the maps. The concept mapping process typically requires participants to brainstorm a large set of statements relevant to a topic of interest, individually sort these statements into groups of similar ones, rate each statement on one or more scales, and interpret a number of visual maps that result from a series of data analyses. Concept mapping methods have been usefully applied in a number of settings and across a variety of complex issues, including assisting students to develop positive concepts of health [18]; identifying multi-level, culturally appropriate smoking cessation strategies for Aboriginal health staff [19]; illustrating conceptual thinking about obesity and bullying prevention strategies in schools [20]; assessing differences in perspectives between administrators/policymakers and those involved in direct practice regarding barriers and facilitators to implementation in large, public mental health service systems [21]; and strategic planning for improving cognitive health [22]. The common factor in these applications of concept mapping is the use of these methods for garnering diverse views on complex topics of interest.

### The research team and ethical review

This research was led by a team from the Australian Prevention Partnership Centre (hereafter the ‘Prevention Centre’) [23]. The research design and conduct was overseen by a Project Steering Committee with expertise in public health, chronic disease prevention and/or methods for concept mapping (SW, WT, AW, AB, CW, NR). The research was reviewed and approved as low-risk by the Administering Institution of the Prevention Centre (R2015/11/06). Participation was anonymous.

### Participant selection

An invitation to participate in the project was sent by email from the Director of the Prevention Centre (AW) to 149 people. The initial email plus up to three reminder emails were sent. Invitees were a purposive sample of individuals with a connection to the Prevention Centre, representing State or Territory and Commonwealth government agencies, funding partners, university-based researchers, Prevention Centre staff and investigators, and individuals working in non-government organisations with a general remit of improving health. A further sample of an additional 20 individuals known to the lead author with expertise or interest in chronic disease prevention were added in the later sorting and rating phases of the research (see description below). While participants were not explicitly asked to forward the email invitation to colleagues, at least one participant is known to have done so. Consequently, the total number of people who were invited to participate in the data collection is likely to be approximately 170.

### Data collection

All data were collected via a web-based process, with analyses conducted and maps produced using the Concept System software specifically designed for this process (Concept System Global Max). Data collection involved two steps, namely (1) statement generation and (2) structuring.

During statement generation, participants ‘brainstormed’ statements [24, 25] guided by the focus prompt: “One specific action we can take in Australia that will improve the prevention of chronic diseases is…” Participants could generate as many statements as possible. They typed statements directly into the web interface, where they could immediately see their ideas along with the ideas from all other participants (the ideas were anonymous and not linked in any way, to maintain the anonymity of contributors). Participants could not challenge or question the statements listed, but could return to the website as often as possible during the brainstorming period to add any additional statements. Following brainstorming, the facilitator (WT) reviewed the statements and edited them for clarity and grammar (but not for content). The Project Steering Committee reviewed and approved the edited statements.

The structuring step involved three distinct tasks, namely demographics of participants, sorting and rating of brainstormed statements. Demographics included general non-identifying information that made it possible to classify participants into subgroups for more detailed analysis. For sorting [24, 26, 27], each participant grouped the statements in a way that made sense to them. The software allowed participants to create, delete and name new groups and to move statements from one group to another. Following grouping, each participant firstly rated the perceived importance of each statement on a 5-point Likert scale, with anchors of 1 = ‘relatively unimportant’ and 5 = ‘extremely important’ (compared with the rest of the statements). Then, participants rated the relative feasibility of each statement on a 5-point Likert scale with anchors of 1 = ‘not at all feasible’ and 5 = ‘very feasible’. These rating scales were selected by the authors based on previous experience, particularly that of WT, in which the scales provided sufficient detail for analyses.

### Analysis

Analyses were conducted using established methods [17] that have been applied and described in detail elsewhere [22]. A series of visual representations of the data were generated through seven steps. First, a unique number was assigned to each statement and an aggregate similarity matrix calculated based on the number of participants who categorised statements similarly. Second, the aggregate similarity matrix was analysed using multidimensional scaling [26] to allocate a place for each statement on an overall map based on an x and y coordinate for each statement. Third, the software combined statements into clusters of similar meaning using hierarchical cluster analysis [28]. Fourth, the software superimposed the results of the hierarchical cluster analysis to create a ‘point map’ in which statements closer to each other are more similar in meaning. A ‘cluster map’ was then generated in which the original statement points were enclosed by polygon-shaped boundaries for the clusters. Fifth, a process of pattern matching [29] was undertaken to develop ‘ladder graphs’ that portrayed the relationships between ratings of importance and feasibility based on the differing demographics of participants. Finally, a ‘go-zone plot’ was generated, providing a visual display of each statement across four quadrants based on ratings of importance and feasibility.

### Interpretation of the concept maps

A preliminary interpretation of results was undertaken by three of the authors (SW, WT and NR). Subsequently, the Project Steering Committee convened to review and interpret the results followed by a group of 20 stakeholders invited to participate based on their interest in and/or influence over national chronic disease prevention efforts. These stakeholders were all part of the initial list of invitees to participate in the project, but it is not known who did or did not contribute to the prior steps of the research. The interpretation sessions for both the Project Steering Committee and stakeholders followed a structured process described in detail elsewhere [16]. In brief, this process involved participants (1) collectively agreeing a short phrase or word to describe or label each cluster of statements (this was achieved through a facilitated process whereby participants were encouraged to openly debate and offer their views) and (2) in turn reviewing the point, cluster, ladder graphs and go-zone maps to see whether they made intuitive sense to them based on their knowledge of the chronic disease prevention efforts and capabilities in the Australian context.

## Results

A total of 78 distinct people participated in the brainstorming. Participation in each stage of the research ranged from 58 to 78 (Box 1). Table 1 summarises participant demographics based on those who completed this stage of data collections.

**Table 1:** Participant demographics

Demographic	Count (%)						Total count (%)
Age	25–34 10 (16)	35–44 13 (21)	45–54 26 (43)	55–64 8 (13)	65+ 4 (7)		61 (100)
Primary field of work	Physical activity 6 (10)	Nutrition 5 (8)	Tobacco 2 (3)	General chronic disease prevention 34 (55)	Other 15 (24)		62 (100)
Type of organisation where they work	Government 18 (29)	Healthcare 3 (5)	NGO or not-for-profit 4 (6)	For profit/private sector 1 (2)	Education and/or research 33 (53)	Other 3 (5)	62 (100)
Main role at work	Senior executive/responsible for strategic direction/chief investigator 18 (29)	Management/senior level planning/senior researcher 20 (32)	Implementation of programmes/researcher 22 (36)	(Programme) practitioners 2 (3)			62 (100)
Area of work	Research 31 (50)	Policy 23 (37)	Practice/service delivery 4 (7)	Other 4 (7)			62 (100)

**Box 1** Participant numbers

**Table:** 

Invited to participate 170 (approximately)	
Step 1. Brainstorming 78 individuals generated statements	
Step 2. Structuring	
82 individuals began sorting of statements 59 completed sorting of statements	
Rated importance of statements	Rated feasibility of statements
66 individuals started rating importance 60 completed rating importance	63 individuals started rating feasibility 58 completed rating feasibility

**Fig. 1:**
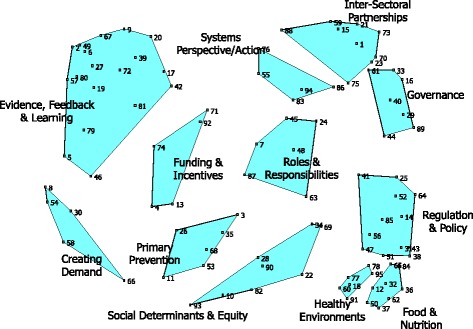
Ninety-five statements organised into 12 clusters

Brainstorming resulted in 131 statements that were edited and synthesised to a final set of 95 statements (Table 2); statements are listed by cluster, with their average importance and average feasibility rating values and sorted within cluster in descending order from most to least important. The two-dimensional scaling analysis yielded a final stress value of 0.28. This value, routinely used to estimate the reliability of concept mapping methods, shows the goodness-of-fit of the map to the sort data and is almost exactly the average stress value found in a meta-analysis of 69 previous concept mapping studies [30]. All possible cluster solutions between 20 and 5 clusters were examined and a final 12-cluster solution was determined to both fit the data well and yield interpretable clusters. The 95 statements, as individual points as well as organised into the labelled clusters, are shown in Fig. 1.

**Table 2:** Statements by cluster with average importance and feasibility values

Inter-sectoral partnerships			
ID	Statement	Importance	Feasibility
88	Develop a deeper understanding of the paradigms of sectors outside of the health sector whose policies and actions influence health and people’s ability to make healthy choices, and who have sway with the community and governments	3.7	3.6
73	Build processes that engage sectors so they have a platform to work together	3.6	3.4
1	Strengthen inter-organisational networks	3.5	3.6
15	Better understand the role, value and impacts of cross-sectoral engagement for prevention	3.5	3.7
75	Invest in State facilitated multi-sector action combined with locally driven approaches, linked to a nationally led framework using the global non-communicable disease targets	3.5	3.1
23	Improve co-ordination of efforts across Federal and State and private health insurance players to avoid duplication of effort	3.5	2.7
70	Engage a more diverse group of stakeholders in public practice	3.4	3.6
59	Develop distributed leadership capacity	3.1	2.8
21	Develop procedures to effectively work through interprofessional power and turf issues, especially to reduce barriers to teamwork and equitable compensation	2.9	2.6
Systems perspective/action			
ID	Statement	Importance	Feasibility
94	Adopt a systems approach to prevention	3.8	3.1
83	Incorporate a systems perspective to understand the preventive health system	3.6	3.3
55	Integrate the consideration of evidence-based health policy and global best practices into local and state government decision-making	3.6	3.3
86	Establish a secure infrastructure to support the delivery of system-wide chronic disease prevention at regional, state and national levels	3.6	2.8
76	Work with relevant university departments to ensure a holistic view of health is included in disciplines that contribute to upstream strategies for chronic disease prevention (e.g. built and natural planning, medical subjects, education, housing, etc.)	3.2	3.5
Governance			
ID	Statement	Importance	Feasibility
16	Adopt a whole-of-government multi-level approach to prevention based on interconnectedness across sectors	4.1	3.1
89	Look beyond the ‘health portfolio’ to ensure all government health polices impact in a positive way on health	3.9	3.3
29	Expect all government agencies to drive changes in their sector that will improve health	3.7	2.6
44	Work towards a health-in-all-policies model	3.7	3.0
33	Incorporate governance to collaboratively address the key levers for change at local, state and national levels	3.4	2.8
61	Develop a governance mechanism that enables resident and community engagement in both co-designing the system and quality improvement	3.2	2.9
40	Organise and coordinate primary care and public health for a defined geographic population	3.1	3.1
Roles and responsibilities			
ID	Statement	Importance	Feasibility
48	Treat prevention the same way as other parts of the healthcare continuum so that it is part of usual health service (embed entities responsible for funding and delivering prevention in Council of Australian Governments agreements)	4.0	2.9
24	Get clarity over whose responsibility it is to fund and deliver prevention	3.9	3.1
45	Promote and facilitate prevention work nationally	3.6	3.6
63	Ensure access to a comprehensive array of services that includes upstream prevention (addressing the socioeconomic determinants of health and behavioural risk factors), clinical prevention and care for those with chronic conditions	3.4	2.7
87	Allow prevention to be treated within the community rating framework in private health insurance (i.e. reinsurable)	3.0	2.9
7	Create competition between Primary Health Networks (PHNs) to improve the health of their population	2.4	3.1
Evidence, feedback and learning			
ID	Statement	Importance	Feasibility
17	Ensure that government preventive health policy and programme decisions are evidence based (e.g. with accountability through scorecards, incentives and open reporting)	4.0	3.2
39	Fund research that is translatable, and that engages policymakers along the way to ensure traction	3.8	3.8
9	Build electronic data systems that are interoperable, allow data sharing, and are useful as feedback loops, and platforms for shared learning and continuous quality improvement, including performance accountability	3.8	3.2
27	Develop robust return on investment data for the main non-communicable disease prevention strategies	3.7	3.5
57	Establish a national health prevention surveillance system linked to national chronic disease and risk factor targets that monitors key non-communicable disease targets (smoking, weight status, physical activity levels, alcohol-related measures)	3.7	3.3
2	Rigorously evaluate prevention initiatives using robust research study designs (e.g. consort criteria)	3.6	3.5
67	Develop an evaluation framework that is consonant with the realities of complex systems and system improvement	3.6	3.5
20	Learn from both global best-practice and domestic successes and failures	3.5	4.1
81	Establish and model the level of investment in strategies that is required to reduce prevalence of major chronic disease risks	3.5	3.5
72	Invest in the health intelligence and knowledge infrastructure to inform our thinking, planning and monitoring	3.5	3.3
80	Develop, communicate and utilise better indicators for health and wellbeing, including return on investment (incorporating health-in-all-policies, triple bottom line policy and economic approaches)	3.5	3.4
42	Review restrictive privacy legislation that prevents data linkages across domains (e.g. Medicare, Pharmaceutical Benefit Scheme and Private Health Insurance) and reduces the ability to engage with the public who have chronic disease	3.5	3.2
6	Build learning systems that allow examination of implementation/adaptation as well as outcomes	3.3	3.4
49	Conduct studies to better define the dose (intensity, duration, reach) required to achieve the Global Targets for non-communicable disease prevention in the Australian context	3.1	3.1
19	Develop a tool box that researchers and policymakers alike can use to measure the impact and potential impact of public health law	3.0	3.6
46	Use a chronic disease prevention score-card report that ranks each PHN area across the country to raise public awareness	2.8	3.3
79	Work with PHNs to create a chronic disease prevention score-card report that ranks each PHN area across the country	2.8	3.4
5	Adopt and promote a national health risk assessment system (like ‘micromorts’) that attributes the health value/cost to specific activities or habits and use this as a way to communicate health risk and change behaviour	2.6	2.7
Funding and Incentives			
ID	Statement	Importance	Feasibility
92	Establish long-term funding mechanisms to support sustainable and on-going work on prevention	4.4	3.0
74	Align financial incentives and supports with strategic objectives and measurable outcomes	3.7	3.1
71	Fund prevention action and research through a national body (e.g. like the recently closed Australian National Preventive Health Agency)	3.6	3.1
13	Incentivise team based, outreach care for patients with complex chronic disease	2.8	3.2
4	Provide capitated funding to GPs based on an enrolled population achieving measured health prevention targets	2.8	2.8
Creating demand			
ID	Statement	Importance	Feasibility
30	Raise the profile of public health with politicians and other decision-makers	4.1	3.6
54	Raise the profile of the benefits of a preventative (rather than curative) focus for the health system	3.9	3.9
8	Improve communication about prevention nationally	3.7	4.2
58	create a sense of urgency in the community about chronic disease	3.6	3.3
66	focus on positive messages about living a fulfilling life	2.7	3.7
Primary Prevention			
ID	Statement	Importance	Feasibility
26	Increase focus on primary prevention	4.1	3.3
3	Put more emphasis on and resources into getting the upstream determinants of health right	4.0	3.0
68	Invest heavily in primary prevention – work with kids and their families	3.6	3.3
11	Take an inclusive vs. normative approach to overweight and obesity (focus on health not weight/specific body mass index) to reduce associated stigma	2.6	3.4
53	Enhance transitional/interstitial care for individuals with chronic disease	2.6	3.1
35	Provide funding for not-for-profit organisations to include healthy activities for children	2.6	3.5
Social determinants and equity			
ID	Statement	Importance	Feasibility
34	Have strategies and plans that address the social determinants of health rather than just refer to them	3.9	3.0
90	Invest in policy innovation that will benefit neglected or marginalised Australian populations who suffer disproportionately – not simply the mainstream	3.9	3.3
28	Ensure a minimum package of basic services for Indigenous Australians	3.8	3.2
93	Emphasise the reduction of inequity	3.8	3.2
82	Better target prevention activities to high risk or vulnerable groups	3.5	3.5
69	Institute a re-distributive health policy that takes account of the unequal distribution of power and resources and contributes to unfair and avoidable health inequities	3.4	2.3
10	Target preconception health to break the cycle of inter-generational risk	3.1	3.3
22	Ensure culturally acceptable, community-led alcohol strategies	3.1	3.2
Healthy environments			
ID	Statement	Importance	Feasibility
91	Encourage incidental physical activity by improving public transport and reducing car density	3.5	3.2
60	Promote physical activity by improving, increasing and joining up walking infrastructure	3.4	3.4
95	Increase opportunity for physical activity through building codes and requirement on property developers	3.4	3.3
78	Actively encourage workplaces through legislation that promotes healthy living	3.3	3.2
18	Provide better access to safe green space	3.3	3.3
77	Improve air quality	2.8	2.5
Food and nutrition			
ID	Statement	Importance	Feasibility
32	Regulate advertising of junk food to children	3.9	3.3
84	Regulate the food environment to limit the fat, sugar and salt content of processed food	3.8	2.9
65	Develop strategies to facilitate/regulate food product reformulation	3.6	3.0
62	Limit access to ‘fast food’ by using zoning laws to reduce the density of fast food outlets	3.3	2.8
12	Reduce fast food serving sizes	3.1	2.7
37	Encourage eating more plant food and trends towards a more vegetarian diet (e.g. through subsidising fruit and vegetables)	3.1	2.7
36	Legislate the labelling of foods containing trans fats	3.0	3.4
50	Limit access to baby formula (e.g. by reducing inappropriate marketing or limiting to prescription only where clinically indicated)	2.2	2.4
Regulation and policy			
ID	Statement	Importance	Feasibility
41	Develop a long-term prevention strategy and funding commitment	4.3	3.3
43	Create and implement urban, regional and rural planning policies that support health-promoting built environments	4.0	3.4
64	Have the Australian Government provide leadership in taking on the food industry through a range of strategies (pricing, marketing, placement, sponsorship) that have been effective in tackling tobacco	3.9	2.8
31	Mandate clear planning mechanisms so our built environment supports physical activity and access to healthy food	3.9	3.2
47	Significantly shift the balance of transport investment towards active travel modes (cycling, walking, public transport)	3.9	2.8
51	Make greater use of regulation and taxation in prevention (e.g. of junk food and sugar-sweetened drinks, introduce an alcohol floor price)	3.8	3.1
38	Make inclusion of active community facilities mandatory for all new town planning and developments	3.7	3.3
56	Use fiscal measures (e.g. taxation) to discourage unhealthy behaviours and encourage healthy ones	3.7	3.0
25	Mandate (legislate) a system of health impact assessments at all levels of government that require legislation and fiscal initiatives to be assessed for their impact on chronic disease, in the same vein as environmental assessments	3.5	2.6
52	Develop a national nutrition policy	3.2	3.8
85	Include health impact analysis and strategies to minimise negative health impact of liquor licensing applications and fast food registrations	3.2	3.2
14	Work with suppliers to impact change, rather than taxing soft drinks	2.3	2.7

**Fig. 2:**
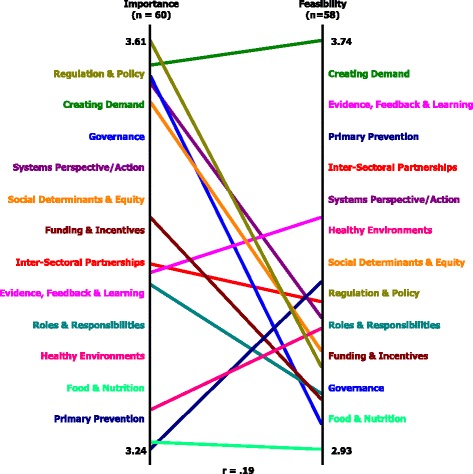
General pattern match of importance and feasibility

The general pattern match (Fig. 2) illustrates how participants rated the importance of certain actions relative to the perceived feasibility of those actions. Several features are notable. The feasibility rating is a skewed distribution with all of the clusters, save one, in the lower half of the axis, meaning participants viewed many of the actions as important but not particularly feasible. The cluster *Creating Demand* is second highest in importance and by far the highest in feasibility. The *Food & Nutrition* cluster is near the bottom on both importance and feasibility. Several of the clusters (*Regulation & Policy*, *Governance* and *Systems Perspective/Action*) are among the highest in importance; however, they are considerably lower in feasibility.

In terms of variations on the general pattern match, a comparison of importance ratings across the major primary fields in which respondents identified themselves, identified several notable points (Additional file 1). Those who identified themselves as ‘General Prevention’ rated the cluster *Systems Perspective/Action* about mid-level in importance, whereas the other two groups – ‘Nutrition, Physical Activity and Tobacco’ and ‘Other’ – rated it highest. Similarly, those who identified themselves as ‘General Prevention’ rated the cluster *Healthy Environments* lowest in importance, whereas the other two groups rated it near the median. In contrast, those in ‘Nutrition, Physical Activity and Tobacco’ rated *Inter-Sectoral Partnerships* lowest in importance, while the other two groups rated it nearer the median.

Similar variations were evident in the pattern matches of importance by self-described respondent organisational type (Additional file 2) as well as main role (Additional file 3). The cluster *Creating Demand* was deemed highest in importance for participants from both ‘Educational/Research’ and ‘Other’ organisations but was considerably lower for those from ‘Government’ who rated *Regulation & Policy* and *Systems Perspective/Action* highest. Those whose role was to ‘Implement’ saw *Evidence, Feedback & Learning* and *Funding & Incentives* as less important than their ‘Management’ or ‘Executive’ colleagues. Finally, there was some evidence that ‘Executives’ saw *Social Determinants & Equity*, *Creating Demand* and *Roles & Responsibilities* as relatively less important than the other groups did.

**Fig. 3:**
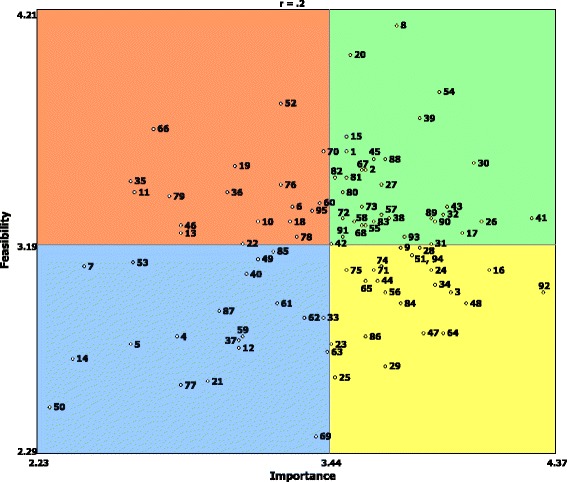
Go-zone plot for all statements

The go-zone plot (Fig. 3) shows statements by ratings of importance and feasibility. The top right quadrant contains the statements perceived by respondents to be most important and most feasible. All actions identified in this quadrant are presented in Box 2. The actions in this quadrant were: “raise the profile of the benefits of a preventative (rather than curative) focus for the health system” (statement number 54); “raise the profile of public health with politicians and other decision-makers” (30); “fund research that is translatable, and that engages policymakers along the way to ensure traction” (39); “improve communication about prevention nationally” (8); “learn from both global best-practice and domestic successes and failures” (20); “increase focus on primary prevention” (26); and “develop a long-term prevention strategy and funding commitment” (41).

**Box 2** Statements rated highest in both importance and feasibility by cluster

**Table:** 

**Inter-Sectoral Partnerships**
Strengthen inter-organisational networks (statement number 1) Better understand the role, value and impacts of cross-sectoral engagement for prevention (15) Develop a deeper understanding of the paradigms of sectors outside of the health sector whose policies and actions influence health and people’s ability to make healthy choices, and who have sway with the community and governments (88) Build processes that engage sectors so they have a platform to work together (73)
**Systems Perspective/Action**
Incorporate a systems perspective to understand the preventive health system (83) Integrate the consideration of evidence-based health policy and global best practices into local and state government decision-making (55)
**Governance**
Look beyond the ‘health portfolio’ to ensure all government health polices impact in a positive way on health (89)
**Roles and Responsibilities**
Promote and facilitate prevention work nationally (45)
**Evidence, Feedback and Learning**
Learn from both global best-practice and domestic successes and failures (20) Fund research that is translatable, and that engages policymakers along the way to ensure traction (39) Rigorously evaluate prevention initiatives using robust research study designs (e.g. consort criteria) (2) Develop an evaluation framework that is consonant with the realities of complex systems and system improvement (67) Develop, communicate and utilise better indicators for health and wellbeing including return on investment (incorporating health-in-all-policies, triple bottom line policy and economic approaches) (80) Establish and model the level of investment in strategies that is required to reduce prevalence of major chronic disease risks (81) Invest in the health intelligence and knowledge infrastructure to inform our thinking, planning and monitoring (72) Develop robust return on investment data for the main non-communicable disease prevention strategies (27) Establish a national health prevention surveillance system linked to national chronic disease and risk factor targets that monitors key non-communicable disease targets (smoking, weight status, physical activity levels, alcohol-related measures) (57) Ensure that government preventive health policy and programme decisions are evidence based (e.g. with accountability through scorecards, incentives and open reporting) (17)
**Creating Demand**
Raise the profile of the benefits of a preventative (rather than curative) focus for the health system (54) Raise the profile of public health with politicians and other decision-makers (30) Improve communication about prevention nationally (8) Create a sense of urgency in the community about chronic disease (58)
**Primary Prevention**
Increase focus on primary prevention (26) Invest heavily in primary prevention – work with kids and their families (68)
**Social Determinants and Equity**
Better target prevention activities to high risk or vulnerable groups (82) Emphasise the reduction of inequity (93) Invest in policy innovation that will benefit neglected or marginalised Australian populations who suffer disproportionately – not simply the mainstream (90)
**Healthy Environments**
Encourage incidental physical activity by improving public transport and reducing car density (91)
**Food and Nutrition**
Regulate advertising of junk food to children (32)
**Regulation and Policy**
Develop a long-term prevention strategy and funding commitment (41) Make inclusion of active community facilities mandatory for all new town planning and developments (38) Create and implement urban, regional and rural planning policies that support health-promoting built environments (43)

Interestingly, no statements within the cluster *Funding & Incentives* were rated as both important and feasible. However, three of the five statements in this cluster, namely “establish long-term funding mechanisms to support sustainable and on-going work on prevention” (92), “align financial incentives and supports with strategic objectives and measurable outcomes” (74) and “fund prevention action and research through a national body (e.g. like the recently closed Australian National Preventive Health Agency)” (71), were rated on average by the group to be important but not feasible. Further, five actions were rated amongst the highest in perceived importance, yet were below the average in terms of perceived feasibility; these actions were “establish long-term funding mechanisms to support sustainable and on-going work on prevention” (92), “adopt a whole-of-government multi-level approach to prevention based on interconnectedness across sectors” (16), “treat prevention the same way as other parts of the healthcare continuum so that it is part of usual health service” (48), “put more emphasis on and resources into getting the upstream determinants of health right” (3) and “have the Australian Government provide leadership in taking on the food industry through a range of strategies (pricing, marketing, placement, sponsorship) that have been effective in tackling tobacco” (64).

## Discussion

This research involved structured activities and advanced multivariate statistical analyses using concept mapping methods to identify priority actions for chronic disease prevention in Australia from the viewpoint of individuals with experience in and knowledge of the Australian chronic disease prevention context.

A total of 95 specific chronic disease prevention actions were identified by participants across 12 clusters, namely (1) Inter-Sectoral Partnerships; (2) Systems Perspective/Action; (3) Governance; (4) Roles and Responsibilities; (5) Evidence, Feedback and Learning; (6) Funding and Incentive; (7) Creating Demand; (8) Primary Prevention; (9) Social Determinants and Equity; (10) Healthy Environments; (11) Food and Nutrition; and (12) Regulation and Policy. Explicitly surfacing and labelling these clusters creates an organising structure for a future national chronic disease prevention strategy, that has status and credibility amongst key actors.

Of the 95 actions, those viewed overall by participants to be the most important and feasible were refocusing the health system to prevention over cure, raising the profile of public health with health decision-makers, funding policy- and practice-relevant research, improving communication about prevention, learning from both global best-practice and domestic successes and failures, increasing the focus on primary prevention, and developing a long-term prevention strategy with an explicit funding commitment. The specificity of actions identified in this research, and the subsequent collective prioritisation by participants, is a unique and potentially powerful supplement for future national policy. Arguably, through this research we have been able to go beyond the generalities often seen in national guiding documents, to explicitly identify specific actions that could be operationalised for national policy.

Reassuringly, there are a number of areas of concordance between the action areas identified in our research and those proposed in a number of the national strategy and policy reform documents for Australia mentioned previously. For example, notions of integrated multidisciplinary action and strategic partnerships are central to the 2005 National Chronic Disease Strategy, the 2008 National Partnership Agreement on Preventive Health as well as the 2009 National Preventive Health Strategy [10–12]. Similarly, in each of these agenda-setting documents, the integral role of primary healthcare in prevention is emphasised. The need for evidence, data, monitoring and surveillance as well as ensuring equity and addressing the social determinants of health is also stated. Calls for responsive regulation [12] are also made. Further, in the 2009 National Partnership Agreement on Preventive Health, an explicit commitment of funding and incentives is made [11]. Importantly, approaches of intersectoral action and support for evidence-based strategies are also identified as overarching principles for the WHO Global Action Plan [1].

Notably, this research also highlighted a number of areas for action that we believe more fully recognise the complexity and systemic nature of chronic diseases noted by others [31]. WHO’s building blocks for health systems, for example, propose six components for health systems strengthening amongst which health financing, leadership and governance are featured as they are in our research [32]. Similarly, the frequently cited Foresight Obesity System Map [33] shares similarities with our findings, with both the food and activity environments recognised in the Foresight Obesity System Map akin to our action clusters of Food and Nutrition and Healthy Environments.

It is difficult in the Australian federated government system and with governments facing re-election every 3–4 years to maintain consistency and persistency of policy and action for the durations necessary to achieve the changes required to prevent chronic disease. It is probably not surprising that policies and strategies are re-invented or re-purposed regularly. Equally, it is not surprising the emphasis of different policies and strategies varies given the political sensitivity associated with some of the changes required to achieve effective preventive action for the most common chronic diseases, for example, in control of advertising of harmful products such as tobacco, alcohol and energy dense food. This project confirms a consistency of views among experts and practitioners about the approaches that need to be taken in chronic disease policy and strategies which should inform new policy and strategy.

There are two key messages from this research. Firstly, preventing chronic diseases and their risk factors will require at-scale, multi-component, multi-stakeholder action and cooperation. Our research signals 12 broad areas for inter-sectoral action, together with indicators for more specific and possibly more immediate national action. Secondly, transformative improvements to the prevention of chronic disease necessitate a paradigm shift in how we approach and invest in prevention. Our research points to a number of areas for proposed action that participants rated as important, yet they did not see them as being feasible in the current context. These actions focussed broadly on a whole-of-government response, leadership in regulations and policies, funding and incentives for prevention efforts, making prevention a mainstream part of healthcare services and addressing the broader social determinants of health.

We note that, whilst participant burden was minimal, participants did need to make a commitment to completing the set tasks. We have received anecdotal feedback that, for some, participation was satisfying and interesting, yet others found the process a little cumbersome and the data collection requirements counterintuitive. Participation numbers for the brainstorming, sorting, importance rating and feasibility rating were 78, 59, 60 and 58, respectively, suggesting caution be taken in particular when interpreting the pattern matches as it is unclear whether and, if so, in what way these results would differ amongst a larger group of respondents. Finally, we recognise that the participants in this research were most likely interested in or indeed advocates for prevention and hence their views may not represent all people who have a role (or potential role) in the prevention of chronic disease across Australia. We recommend additional research be undertaken, most likely a qualitative group process, to allow for a more in-depth examination across all sectors that have a role in impacting chronic disease prevention. This will allow further exploration of the actions as well as foster further momentum for system change and collaborative efforts.

## Conclusions

Through well established, rigorous concept mapping methods, this research has surfaced and synthesised expert opinion to create a prioritised set of specific actions for chronic disease prevention in Australia across 12 theme areas. It is recommended that the data and maps generated by this process be used as a reference point for stimulating dialogue and engagement amongst the people and organisations who collectively have both a stake in and power to improve chronic disease prevention in Australia. Arguably, the data from this research could help in informing a shared vision and common agenda for specific, prioritised, coordinated, national prevention actions – both in the immediate and longer term.

## Additional files

Additional file 1: Importance by Primary Field. (DOCX 22 kb)

Additional file 2: Importance by Organisation. (DOCX 22 kb)

Additional file 3: Importance by Main Role. (DOCX 22 kb)
